# Supplementary material for: Genome-Wide Association and Prediction of Traits Related to Salt Tolerance in Autotetraploid Alfalfa (Medicago sativa L.)
Source: Int J Mol Sci. 2020 May 9;21(9):3361. doi: 10.3390/ijms21093361 (PMC7247575; doi:10.3390/ijms21093361)
Supplement: Supplementary file 1 [file ijms-21-03361-s001.pdf]

Supplementary data.

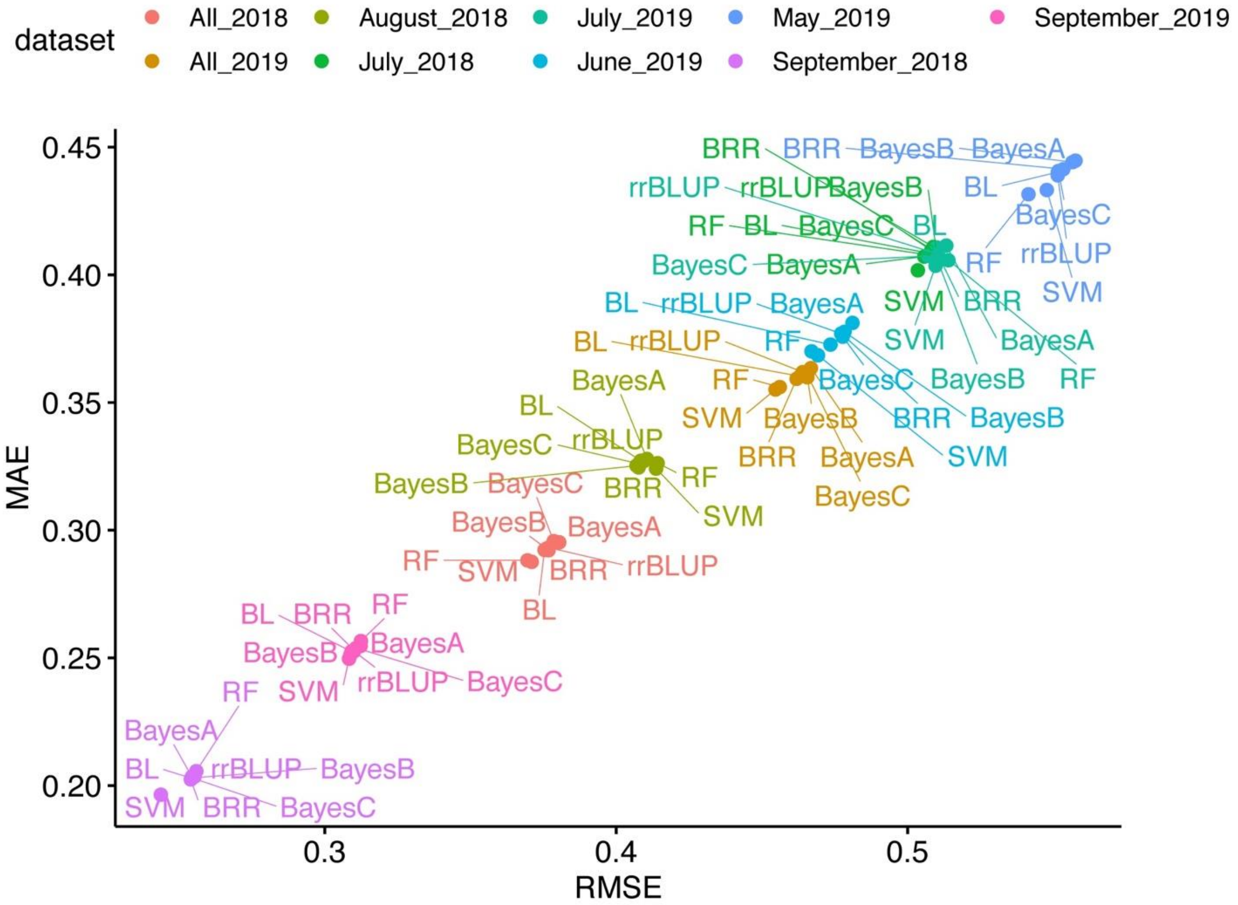

Figure S1. Correlation scatter plot between mean absolute error (MAE) and root mean squared error (RMSE) values of GS results for eight different models in yield.

Table S1. Hyperparameters autoadjusted by caret R package in random forest (RF) and support vector machine (SVM) models to obtain the lowest value of RMSE. The values were obtained of model with 10-fold cross validation (10CV) and with 10% of the data to test the model (10%). Hyperparameters tuned in SVM were sigma = defining how far is the influence of training predictors in the regression and cost (C) = $\{0.25, 0.5, 1.0\}$ were used to control the trade-off between smooth decision boundary (hyperplane) that classifies the training predictors correctly. Hyperparameters tuned in RF were mtry = number of predictors (SNPs) randomly selected at each tree node $\{2, 116, 6832\}$ and split rule = splitting rule to use during tree construction for regression which can be "variance" or "extra-trees".

		SVM		RF	
Dataset	Testing	sigma	cost	mtry	split_rule
July_2018	10%	1.21E-04	1	6832	variance
	10CV	1.23E-04	1	6832	extratrees
August_2018	10%	1.21E-04	1	6832	extratrees
	10CV	1.20E-04	1	116	variance
September_2018	10%	9.82E-05	1	6832	variance
	10CV	9.86E-05	1	6832	extratrees
All_2018	10%	1.21E-04	1	6832	variance
	10CV	1.23E-04	1	6832	variance
May_2019	10%	1.00E-04	0.25	6832	variance
	10CV	1.01E-04	0.25	6832	extratrees
June_2019	10%	1.25E-04	0.5	116	extratrees
	10CV	1.22E-04	1	6832	variance
July_2019	10%	1.26E-04	1	6832	extratrees
	10CV	1.22E-04	1	116	variance
September_2019	10%	1.23E-04	1	6832	variance
	10CV	1.22E-04	1	6832	variance
All_2019	10%	1.01E-04	0.25	6832	variance
	10CV	1.22E-04	0.25	6832	extratrees

Table S2. BLUEs values calculated for yield during three harvest in 2018, four harvest in 2019, all 2018 and all 2019 in WA and vigour (V) under salt stress in two locations WA and UT. NA correspond to missing plants during the experiment or non-tested.

Line	All_2018	Jul_2018	Aug_2018	Sep_2018	All_2019	May_2019	Jun_2019	Jul_2019	Sep_2019	V_UT	V_WA
1_1_S283	0.469	0.708	0.366	0.052	0.412	0.511	0.065	-0.184	-0.086	4.0	4.5
1_2_S295	1.159	1.756	1.263	0.469	1.060	0.783	0.846	0.770	0.659	4.0	3.5
1_3_S307	0.812	1.178	0.944	0.365	0.957	1.167	0.827	0.431	0.315	4.0	3.5
1_4_S319	0.599	1.083	0.639	0.300	0.867	0.999	0.715	0.625	0.279	4.0	4.0
1_5_S331	0.934	1.301	1.074	0.576	0.779	1.272	0.945	0.739	0.378	4.0	4.0
1_6_S343	0.943	1.400	1.060	0.300	1.050	1.593	1.038	0.846	0.457	4.0	4.5
1_7_S284	0.854	1.530	1.034	0.290	0.914	1.280	1.377	0.913	0.450	5.0	5.0
1_8_S296	1.057	1.877	1.160	0.344	0.976	1.897	1.248	0.934	0.336	3.0	3.5
10_2_S339	0.772	1.004	0.752	0.245	0.945	1.347	0.892	0.627	0.283	3.0	3.0
10_3_S351	0.245	0.627	0.321	0.126	0.495	0.704	0.508	0.355	0.224	3.0	2.5
10_6_S362	0.940	1.686	0.916	0.416	0.681	0.750	0.502	0.304	0.236	3.0	3.5
10_7_S373	1.005	1.038	1.247	0.573	1.374	1.075	1.180	1.180	0.868	4.0	4.0
10_8_S292	1.099	1.672	1.456	0.738	1.112	1.425	1.140	1.445	1.105	3.0	4.0
11_1_S304	0.655	0.996	0.987	0.286	0.305	0.900	0.599	0.650	0.341	2.0	2.5
11_2_S316	1.439	1.837	1.622	0.690	1.484	1.944	1.445	1.416	0.938	4.0	4.5
11_3_S328	1.344	1.936	1.235	0.753	1.007	1.206	0.830	0.794	0.919	3.0	3.5
11_4_S340	1.387	1.835	1.475	0.865	0.550	0.516	0.395	0.377	0.306	4.0	4.5
11_5_S352	0.487	0.637	0.453	0.299	0.410	0.731	0.588	0.438	0.181	4.0	3.0
11_6_S363	0.537	0.659	0.635	0.366	0.195	0.696	0.544	0.304	0.298	2.0	2.5
11_7_S374	1.126	1.379	1.042	0.792	1.027	1.419	1.082	1.379	0.885	4.0	4.0
11_8_S293	1.541	2.035	1.673	0.854	1.463	2.062	1.558	1.377	0.785	5.0	5.0
12_1_S305	1.283	1.587	1.438	0.541	1.232	1.385	1.458	1.228	0.756	5.0	4.5
12_2_S317	1.107	1.498	1.238	0.607	1.124	1.508	1.282	1.435	1.015	5.0	4.5
12_3_S329	0.709	1.108	0.758	0.384	0.841	1.183	0.858	0.473	0.238	5.0	5.0
12_4_S341	1.389	1.666	1.278	0.810	1.406	1.683	1.814	1.091	0.842	4.0	3.5
12_5_S353	1.074	1.362	1.127	0.337	1.472	1.828	1.374	1.205	0.659	4.0	4.0
12_6_S364	1.015	1.028	0.848	0.594	1.215	1.360	0.859	1.044	0.861	5.0	4.5
12_7_S375	1.277	1.790	0.958	0.818	1.332	1.504	1.349	1.440	0.661	4.0	4.0
12_8_S294	1.118	1.599	1.241	0.686	0.799	0.977	0.721	0.715	0.402	4.0	4.0
13_1_S306	1.495	2.167	1.867	0.918	1.684	2.560	1.933	1.692	1.198	5.0	5.0

13_2_S318	0.421	0.983	0.622	0.392	0.395	0.821	0.747	0.710	0.149	2.0	3.0
13_3_S330	2.236	2.595	2.047	1.513	1.779	2.752	1.889	1.659	0.978	5.0	4.5
13_4_S342	1.804	2.398	1.988	1.040	1.539	2.007	1.637	1.904	1.219	5.0	5.0
13_5_S354	1.118	1.296	1.154	0.682	0.542	1.148	0.816	0.755	0.568	3.0	3.0
13_6_S365	1.595	2.023	1.671	0.853	1.059	1.688	1.350	1.171	0.810	5.0	5.0
13_7_S376	0.941	1.336	0.997	0.653	0.765	1.409	1.378	1.328	0.281	4.0	4.5
13_8_S377	0.568	0.947	0.762	0.236	0.140	0.709	0.403	0.383	0.172	4.0	4.0
14_1_S388	0.889	1.163	1.008	0.499	1.071	1.329	1.117	1.071	0.730	3.0	3.0
14_2_S399	0.493	0.531	0.385	0.166	0.282	1.013	0.510	0.447	0.245	3.0	3.0
14_4_S411	0.572	0.730	0.568	0.213	0.298	0.777	0.343	0.390	0.284	4.0	4.5
14_5_S423	0.915	1.340	0.929	0.362	0.844	1.368	0.536	0.493	0.209	2.0	2.5
14_6_S435	0.572	0.619	0.553	0.240	0.455	0.726	0.590	0.536	0.357	3.0	3.5
14_8_S447	0.398	0.725	0.615	0.143	0.385	0.549	0.499	0.307	0.065	3.0	3.0
15_1_S459	0.964	1.009	0.922	0.388	0.499	0.580	0.379	0.577	0.580	4.0	3.5
15_2_S400	1.070	1.075	0.846	0.552	0.707	0.618	0.461	0.507	0.485	4.0	4.5
15_4_S412	2.174	2.557	2.475	1.426	1.978	2.469	2.029	2.269	1.245	4.0	4.5
15_5_S424	0.827	1.469	1.146	0.353	0.583	1.077	0.680	1.122	0.682	3.0	2.5
15_7_S436	1.249	1.699	1.388	0.936	0.838	0.772	0.661	0.758	0.583	5.0	5.0
15_8_S448	1.856	2.163	2.203	1.250	1.248	1.464	1.353	1.719	1.587	3.0	3.5
16_1_S460	1.247	1.887	1.316	0.458	NA	NA	NA	NA	NA	4.0	4.0
16_2_S378	1.254	1.667	1.358	0.694	1.403	1.542	1.500	1.573	1.015	4.0	4.0
16_3_S389	0.815	1.422	1.026	0.226	1.102	1.661	1.226	0.890	0.286	3.0	3.5
16_4_S401	2.262	3.445	2.234	1.069	1.634	2.689	2.124	1.319	0.364	4.0	4.5
16_5_S413	0.881	1.623	1.010	0.590	1.088	1.435	1.315	1.198	0.396	3.0	3.5
16_6_S425	0.969	2.093	1.081	0.399	0.678	1.099	0.645	0.559	0.605	3.0	3.0
16_7_S437	0.694	1.094	0.788	0.465	0.145	0.516	0.089	0.059	0.002	4.0	3.5
16_8_S449	0.993	1.678	0.957	0.671	0.710	1.308	0.770	0.774	0.295	4.0	4.0
17_1_S461	0.874	1.363	0.873	0.499	0.939	1.250	1.159	1.099	0.699	3.0	3.0
17_2_S379	1.571	2.413	1.567	0.964	1.483	1.851	1.273	1.328	1.060	2.0	3.5
17_3_S390	0.595	0.983	0.599	0.302	0.853	1.511	0.969	0.773	0.348	2.0	3.0
17_4_S402	1.317	1.733	0.990	0.460	0.689	1.142	1.030	0.834	0.543	4.0	4.0
17_5_S414	1.024	1.733	0.844	0.356	0.405	0.553	0.284	0.250	0.280	4.0	4.0
17_6_S426	1.162	1.456	1.043	0.555	0.683	0.323	0.683	0.773	0.745	5.0	4.5
17_7_S438	1.134	1.731	0.952	0.599	1.058	1.664	0.945	0.876	0.563	4.0	4.0

17_8_S450	1.445	1.995	1.690	0.869	1.220	1.879	1.884	1.828	1.097	1.0	3.0
18_1_S462	1.230	1.912	1.339	0.592	0.838	1.553	1.290	1.604	0.649	4.0	4.5
18_2_S380	1.342	1.974	1.523	0.630	1.035	1.239	1.269	1.440	0.882	3.0	3.5
18_3_S391	0.958	1.881	1.299	0.224	0.594	1.547	1.236	0.753	0.349	4.0	4.5
18_4_S403	0.590	0.806	0.854	0.227	0.649	1.152	0.815	0.702	0.429	3.0	3.5
18_5_S415	1.115	1.304	1.153	0.599	0.060	0.183	-0.050	NA	NA	3.0	3.5
18_6_S427	0.910	1.143	0.831	0.198	1.037	1.430	0.931	0.721	0.340	2.0	3.0
18_7_S439	1.393	1.620	1.268	0.634	1.364	1.456	1.089	1.046	0.804	4.0	4.5
18_8_S451	0.612	0.917	0.644	0.288	0.490	0.205	0.103	0.040	0.075	3.0	3.5
19_1_S463	1.448	1.787	1.455	1.006	0.944	1.330	0.813	0.625	0.868	2.0	3.0
19_2_S381	1.609	2.342	1.746	1.068	0.667	0.923	0.568	0.796	0.623	5.0	5.0
19_3_S392	0.990	1.871	1.411	0.791	1.226	1.559	1.076	0.892	0.843	4.0	3.5
19_4_S404	1.170	1.590	1.600	0.953	1.226	1.577	1.210	0.910	0.696	3.0	3.0
19_5_S416	0.539	1.039	1.270	0.205	0.403	0.679	0.542	0.356	0.209	3.0	3.0
19_7_S428	1.089	1.828	1.583	0.437	0.048	0.068	-0.176	NA	NA	5.0	4.5
19_8_S440	1.290	1.326	1.159	0.778	0.638	0.919	0.895	0.846	0.137	4.0	4.0
2_1_S308	1.494	1.680	1.566	0.937	0.986	1.279	0.897	0.696	0.446	4.0	4.5
2_2_S320	0.953	1.284	1.051	0.605	0.708	0.812	0.753	0.410	0.544	4.0	4.0
2_3_S332	0.867	1.286	1.000	0.531	0.635	0.729	0.545	0.436	0.223	4.0	4.0
2_4_S344	0.999	1.411	0.852	0.494	0.914	1.124	0.964	0.774	0.635	4.0	4.0
2_5_S355	0.851	0.944	0.731	0.453	0.327	0.312	0.527	0.617	0.750	5.0	4.5
2_6_S366	0.882	0.853	0.730	0.622	1.038	1.022	0.737	0.489	0.588	5.0	5.0
2_7_S285	1.283	1.625	1.478	0.940	1.136	1.192	1.202	0.923	1.007	5.0	5.0
2_8_S297	0.876	1.243	1.383	0.570	1.078	1.775	1.407	1.054	0.766	4.0	4.0
20_1_S452	1.697	2.627	1.901	0.895	1.625	1.938	1.458	1.474	0.937	5.0	5.0
20_2_S464	1.029	1.472	0.878	0.497	0.847	1.163	0.809	0.768	0.511	5.0	4.5
20_3_S382	0.984	1.494	1.076	0.516	0.463	1.029	0.643	0.788	0.357	5.0	5.0
20_4_S393	1.122	1.814	1.391	0.871	0.132	0.806	0.732	0.796	0.424	4.0	4.0
20_5_S405	2.035	2.570	2.109	1.071	1.362	2.534	1.625	1.483	0.823	5.0	5.0
20_6_S417	1.188	1.785	1.302	0.631	0.971	1.752	1.382	0.855	0.468	5.0	4.5
20_7_S429	1.012	1.360	1.234	0.560	0.019	0.676	0.494	0.234	0.366	5.0	4.0
20_8_S441	1.542	2.197	1.505	0.820	1.073	1.094	1.466	1.884	0.812	.	5.0
21_1_S453	1.107	1.363	1.095	0.692	0.762	1.249	0.704	1.353	1.170	5.0	4.5
21_2_S465	0.362	0.441	0.566	0.490	NA	NA	NA	NA	NA	4.0	3.0

21_3_S383	1.815	2.243	1.767	1.127	2.087	1.833	3.132	2.980	1.714	4.0	4.0
21_4_S394	2.157	2.946	2.273	1.267	1.439	1.638	1.031	2.362	1.036	5.0	5.0
21_5_S406	1.637	2.640	1.906	0.938	2.005	2.564	1.813	1.912	1.014	4.0	4.5
21_6_S418	1.127	1.761	1.588	0.690	1.870	1.308	1.065	2.331	1.808	5.0	5.0
21_7_S430	1.348	2.218	1.728	0.810	0.646	0.970	0.923	0.822	0.203	5.0	4.0
21_8_S442	0.426	0.887	0.451	0.228	0.728	0.728	NA	NA	NA	3.0	4.0
22_2_S454	0.910	1.234	0.954	0.270	0.982	0.853	0.998	0.818	0.496	3.0	2.5
22_3_S466	1.275	2.468	1.110	0.817	1.057	1.461	1.348	1.254	0.743	4.0	4.0
22_4_S384	1.672	2.266	1.425	0.911	1.308	1.669	1.451	1.275	0.655	4.0	4.5
22_5_S395	1.615	2.329	1.815	1.164	1.305	1.415	1.385	1.633	0.933	3.0	3.0
22_6_S407	1.740	2.229	1.872	1.276	1.541	2.076	1.779	1.689	1.101	4.0	4.5
22_7_S419	0.723	1.032	0.811	0.484	0.290	0.615	0.599	0.458	0.307	3.0	3.5
22_8_S431	0.851	1.272	0.644	0.557	0.739	1.458	1.064	0.863	0.551	4.0	4.5
23_1_S443	0.972	1.311	1.138	0.525	0.333	0.641	1.073	0.689	0.334	5.0	4.5
23_2_S455	0.970	1.185	0.922	0.329	0.954	1.431	1.122	0.768	0.309	5.0	4.5
23_3_S467	1.208	1.681	1.254	0.522	1.013	1.483	0.917	0.334	0.123	4.0	4.5
23_4_S385	1.755	2.254	1.545	0.908	1.365	1.472	1.456	1.217	0.643	5.0	5.0
23_5_S396	1.153	1.660	1.490	0.728	0.642	1.363	0.826	0.780	0.623	5.0	4.5
23_6_S408	1.080	1.520	1.189	0.579	0.939	1.577	1.110	0.749	0.375	4.0	3.5
23_7_S420	0.760	1.231	0.951	0.268	0.923	1.397	1.043	1.019	0.700	5.0	4.0
23_8_S432	1.599	1.828	1.735	1.093	1.304	1.634	1.190	1.348	1.010	3.0	3.0
24_1_S444	0.996	1.165	1.432	0.352	0.713	1.171	0.656	0.328	0.352	4.0	3.5
24_2_S456	0.698	0.563	0.608	0.524	0.481	0.513	0.518	0.833	0.660	2.0	2.5
24_3_S468	1.195	1.752	1.330	0.573	1.033	1.274	1.135	1.742	0.977	5.0	4.0
24_4_S386	0.858	0.994	0.823	0.577	0.560	0.916	0.475	0.550	0.340	4.0	3.5
24_5_S397	0.715	0.533	0.667	0.167	NA	NA	NA	NA	NA	3.0	3.0
24_6_S409	0.864	1.164	0.976	0.387	0.474	0.621	0.500	0.484	0.383	3.0	3.0
24_7_S421	0.827	1.462	0.921	0.432	0.502	0.830	0.628	0.567	0.424	5.0	4.5
24_8_S433	1.253	1.819	1.166	0.656	0.894	1.782	0.729	0.733	0.260	3.0	3.5
26b_1_S434	1.224	1.165	2.417	0.738	-0.813	0.435	NA	NA	NA	NA	NA
26b_2_S446	0.746	1.401	0.693	0.537	0.786	0.967	0.943	0.578	0.204	NA	NA
26b_3_S458	0.644	0.233	1.215	0.264	0.436	0.791	0.479	0.662	0.457	NA	NA
26b_4_S470	0.985	1.361	1.215	0.818	1.065	1.340	1.042	2.413	0.697	NA	NA
26b_5_S471	0.614	0.945	0.396	0.322	1.733	1.513	1.659	1.544	0.975	NA	NA

26b_6_S483	1.054	1.636	1.111	0.711	1.210	1.269	1.065	1.013	0.605	NA	NA
26b_7_S495	0.808	0.901	0.772	0.446	0.289	0.694	0.170	NA	NA	NA	NA
26b_8_S506	1.403	1.309	1.395	0.996	0.869	0.509	NA	NA	NA	NA	NA
27_1_S517	0.447	0.694	0.606	0.426	0.212	0.370	0.567	0.489	0.215	5.0	4.5
27_3_S529	1.781	2.575	1.878	1.107	1.412	2.023	1.800	1.618	1.137	4.0	4.0
27_4_S541	1.675	2.120	1.680	1.168	1.123	1.735	1.427	1.455	0.592	4.0	3.5
27_7_S553	1.121	1.465	1.281	0.606	1.075	1.154	1.310	1.177	0.860	4.0	3.5
27_8_S472	1.094	1.553	1.267	0.652	1.008	1.188	1.322	1.165	0.593	5.0	4.0
28_1_S484	1.268	1.862	1.471	0.679	0.736	0.895	0.777	0.629	0.653	5.0	4.5
28_2_S518	0.866	0.950	0.976	0.572	0.336	0.436	NA	NA	NA	5.0	4.5
28_3_S530	0.767	1.079	0.514	1.199	0.485	0.998	0.593	0.064	0.099	4.0	3.0
28_4_S542	1.212	2.138	1.224	0.455	0.496	1.164	0.630	0.357	0.443	5.0	4.5
28_5_S554	1.015	1.471	0.771	0.469	1.034	1.010	0.540	0.305	0.493	5.0	5.0
28_6_S473	1.496	2.069	1.878	0.822	1.055	2.071	1.412	1.619	1.007	4.0	4.0
28_7_S485	1.701	1.915	1.676	0.980	1.092	1.003	1.203	1.261	1.088	4.0	0.0
28_8_S496	1.074	1.644	1.217	0.360	0.788	1.395	1.126	0.953	0.431	4.0	3.5
29_1_S507	1.167	1.715	1.216	0.463	0.847	1.621	0.787	0.422	0.483	4.0	3.5
29_2_S519	0.699	0.995	0.610	0.384	1.190	1.282	1.120	1.010	0.474	3.0	3.0
29_3_S531	1.045	1.431	1.510	0.608	0.475	1.285	0.857	0.431	0.557	4.0	3.5
29_4_S543	1.792	2.541	1.736	0.698	1.462	2.327	2.147	2.408	1.055	4.0	4.0
29_5_S555	1.680	2.328	1.586	0.883	1.383	2.008	1.291	0.957	0.732	4.0	4.0
29_6_S474	1.084	1.376	1.267	0.587	-0.109	0.089	0.274	0.217	NA	3.0	3.0
29_7_S486	1.595	2.271	1.928	0.589	0.731	1.267	1.527	2.199	1.334	4.0	3.5
29_8_S497	0.891	1.003	1.266	0.410	0.646	0.873	1.321	1.260	0.881	5.0	4.0
3_1_S309	1.627	2.151	1.813	0.926	1.769	1.727	1.211	1.256	0.725	4.0	4.0
3_2_S321	1.190	1.735	1.333	0.582	0.778	1.154	0.758	0.809	0.758	2.0	3.5
3_3_S333	0.842	1.140	0.992	0.525	0.753	0.891	0.767	0.942	0.789	2.0	3.0
3_4_S345	1.041	1.726	1.071	0.565	0.937	1.794	1.369	0.955	0.179	4.0	4.0
3_5_S356	1.041	1.224	1.087	0.297	1.043	0.816	0.663	0.599	0.180	4.0	3.0
3_6_S367	0.858	0.969	0.819	0.413	0.632	0.938	0.737	0.435	0.418	4.0	4.0
3_7_S286	0.902	1.448	1.028	0.449	0.566	0.701	0.720	0.375	0.430	5.0	4.5
3_8_S298	0.956	1.526	1.121	0.497	0.285	0.493	0.355	0.356	0.390	3.0	3.0
30_2_S508	0.683	0.838	0.650	0.438	0.689	0.744	0.639	0.909	0.618	4.0	4.0
30_3_S520	1.469	2.138	1.652	1.128	1.435	1.724	1.640	1.665	1.091	3.0	3.0

30_4_S532	1.583	2.214	1.462	0.695	0.790	0.807	0.834	0.453	0.098	3.0	3.5
30_5_S544	2.148	2.452	2.194	1.232	NA	NA	NA	NA	NA	4.0	4.0
30_6_S556	1.203	1.607	1.397	0.643	0.772	1.323	1.238	1.471	0.776	3.0	4.0
30_7_S475	1.027	1.490	1.166	0.758	1.007	1.426	0.598	1.558	0.728	3.0	3.5
30_8_S487	1.168	1.523	1.406	0.760	-0.281	0.401	0.109	-0.134	NA	4.0	4.0
31_1_S498	1.388	2.004	1.699	0.749	0.754	1.253	0.824	0.480	0.351	5.0	5.0
31_2_S509	0.885	1.173	0.874	0.414	0.705	0.663	0.431	1.003	0.468	3.0	3.0
31_3_S521	1.167	1.492	1.144	0.571	1.140	1.428	1.293	1.212	0.854	3.0	3.5
31_4_S533	1.504	2.090	1.179	0.626	0.637	1.156	1.271	1.126	0.558	3.0	3.5
31_5_S545	1.286	1.566	1.373	0.852	0.945	1.190	1.081	1.169	0.634	3.0	3.0
31_6_S557	0.874	1.028	0.862	0.343	0.389	0.658	0.360	0.229	0.117	3.0	3.0
31_7_S476	0.651	0.757	0.745	0.277	0.882	0.897	0.729	0.289	0.175	4.0	4.0
31_8_S488	0.567	0.884	0.514	0.254	0.584	0.719	0.676	0.499	0.343	3.0	3.0
32_1_S499	1.114	1.732	1.192	0.445	1.428	1.636	1.102	0.959	0.702	3.0	3.0
32_2_S510	0.740	1.022	0.978	0.541	0.840	0.814	0.764	0.436	NA	4.0	4.0
32_3_S522	0.879	1.059	0.787	0.444	1.015	0.925	0.985	0.718	0.502	2.0	2.0
32_4_S534	0.975	1.136	1.329	0.478	0.191	0.461	0.425	0.690	0.337	3.0	3.0
32_5_S546	0.884	1.172	0.954	0.392	0.751	0.701	0.547	0.539	0.355	3.0	2.5
32_6_S558	0.981	1.361	1.015	0.551	0.555	0.822	0.896	1.112	0.598	3.0	3.0
32_7_S477	0.712	1.015	0.833	0.301	0.709	0.936	0.844	0.645	0.402	3.0	2.5
32_8_S489	0.738	1.252	0.458	0.507	0.323	0.486	0.394	0.399	0.323	4.0	3.0
33_1_S500	1.127	1.560	1.485	0.569	0.354	1.147	1.044	0.898	0.510	4.0	4.0
33_2_S511	0.727	0.698	0.526	0.165	0.541	0.624	0.577	0.395	0.144	2.0	2.5
33_3_S523	0.893	1.190	0.955	0.300	0.343	0.717	0.870	0.433	0.216	3.0	3.5
33_4_S535	1.014	1.513	1.315	0.699	1.216	1.664	1.374	1.275	0.747	3.0	3.5
33_5_S547	1.414	2.053	1.700	0.804	1.047	2.450	1.772	1.304	0.594	3.0	3.5
33_6_S559	1.018	1.256	1.055	0.440	1.270	1.359	1.465	1.100	0.647	3.0	3.5
33_7_S478	0.545	1.136	0.590	0.220	0.404	1.074	1.102	0.879	0.440	3.0	3.0
33_8_S490	0.886	1.611	0.854	0.292	0.693	1.179	0.918	0.595	0.411	3.0	3.5
34_1_S501	0.496	0.624	0.761	0.350	0.587	0.783	0.522	0.437	0.239	3.0	2.5
34_2_S512	0.827	1.160	0.853	0.157	0.978	1.742	0.650	0.420	0.124	3.0	2.5
34_3_S524	0.670	1.141	1.189	0.597	0.603	0.705	0.811	0.569	0.428	4.0	3.5
34_4_S536	1.626	1.888	2.013	0.946	0.798	0.737	0.663	0.473	0.470	4.0	4.0
34_5_S548	0.895	0.745	0.798	0.426	0.911	0.793	0.758	0.695	0.324	4.0	3.5

34_6_S560	1.037	1.280	1.059	0.621	1.445	1.491	1.386	1.286	1.209	4.0	3.5
34_7_S479	1.251	2.041	1.619	0.470	1.781	1.637	1.603	1.333	0.841	4.0	3.5
34_8_S491	1.223	1.492	1.112	0.347	1.110	2.033	1.089	0.888	0.643	3.0	3.0
35_1_S502	0.912	1.257	0.959	0.653	0.062	0.017	NA	NA	NA	3.0	3.5
35_2_S513	0.477	0.780	0.554	0.410	0.196	0.444	0.489	0.361	0.297	3.0	3.0
35_3_S525	1.544	2.040	1.829	0.960	1.111	1.546	1.331	1.285	0.952	3.0	3.5
35_4_S537	0.816	0.912	1.133	0.478	NA	NA	NA	NA	NA	3.0	3.0
35_5_S549	1.222	1.622	1.391	0.797	0.401	0.697	0.829	0.726	0.883	1.0	2.0
35_6_S561	1.023	1.211	1.077	0.614	0.634	1.269	1.223	1.011	0.218	4.0	3.5
35_7_S480	0.682	1.116	0.458	0.217	0.194	0.507	0.386	0.493	0.162	2.0	2.5
35_8_S492	0.694	0.979	0.811	0.407	0.391	0.818	0.625	0.496	0.308	3.0	3.0
36_1_S503	0.590	0.727	0.831	0.397	0.326	0.297	0.390	0.437	0.285	4.0	4.0
36_2_S514	0.548	1.007	0.534	0.297	0.608	1.049	0.795	0.598	0.151	3.0	3.0
36_3_S526	1.122	1.886	1.130	0.590	0.815	0.867	0.954	1.360	0.655	4.0	3.5
36_4_S538	0.687	1.247	0.950	0.332	0.829	0.552	0.847	0.500	0.547	3.0	3.5
36_5_S550	1.270	2.071	1.085	0.394	0.491	1.056	0.406	0.336	0.146	5.0	4.5
36_6_S562	0.706	1.066	0.804	0.367	0.119	0.453	0.414	0.642	0.444	4.0	3.5
36_7_S481	0.597	1.141	0.841	0.266	0.269	0.803	0.417	0.142	0.287	4.0	4.0
36_8_S493	0.681	1.142	0.874	0.295	0.973	1.425	1.087	1.029	0.870	4.0	4.0
37_1_S504	1.165	1.727	1.448	0.733	0.753	0.789	0.353	0.360	0.334	4.0	3.5
37_2_S515	1.553	1.949	1.575	1.203	1.278	1.166	1.407	1.826	0.762	5.0	5.0
37_4_S527	0.642	0.779	0.667	0.313	0.005	0.398	0.553	0.488	0.537	4.0	3.5
37_5_S539	1.314	1.777	1.413	0.860	0.703	0.989	1.074	1.160	0.558	5.0	4.5
37_6_S551	1.048	1.374	1.181	0.737	0.623	1.262	1.025	1.167	0.639	5.0	4.5
37_7_S563	1.022	1.524	1.320	0.786	0.807	0.899	0.742	0.772	0.519	4.0	4.0
37_8_S482	1.243	1.338	1.242	0.840	0.025	0.296	NA	NA	NA	5.0	5.0
38_1_S494	1.601	2.094	1.727	1.069	0.954	0.561	0.558	0.756	0.572	4.0	4.5
38_3_S505	0.929	1.453	0.945	0.437	0.782	0.831	0.705	0.490	0.354	3.0	3.5
38_4_S516	1.046	1.555	1.047	0.501	0.654	1.096	0.859	0.311	0.439	3.0	3.5
38_5_S528	1.639	2.007	2.042	0.926	1.205	1.757	1.829	1.478	1.168	3.0	3.5
38_6_S540	1.304	2.354	1.231	0.686	1.262	1.851	1.548	1.422	0.888	4.0	4.0
38_7_S552	2.079	3.024	2.140	1.171	2.732	2.837	2.947	2.540	1.235	3.0	3.5
38_8_S564	1.366	2.032	1.368	0.812	0.509	0.552	0.374	0.265	0.244	4.0	5.0
4_1_S310	1.120	1.712	1.437	1.024	0.854	1.170	1.107	1.027	0.753	5.0	3.5

4_2_S322	0.832	1.502	1.018	0.507	-0.126	0.223	0.296	0.170	0.097	.	2.0
4_3_S334	1.237	2.071	1.130	0.614	0.626	0.719	0.992	0.757	0.716	4.0	4.0
4_4_S346	0.751	0.834	0.747	0.511	0.704	0.852	0.549	0.584	0.562	3.0	2.5
4_5_S357	1.130	1.319	1.124	0.432	0.465	0.378	0.599	0.582	0.268	3.0	3.0
4_6_S368	1.035	1.370	1.146	0.575	1.216	1.409	1.465	1.473	0.926	5.0	4.5
4_8_S287	1.645	1.840	1.677	1.269	1.334	1.348	1.380	1.602	1.211	5.0	5.0
5_2_S299	0.917	1.214	1.034	0.521	0.669	1.075	0.816	0.940	0.697	4.0	4.0
5_4_S311	1.180	1.440	1.268	0.891	1.203	1.406	0.994	1.509	1.037	3.0	3.5
5_6_S323	0.831	0.917	0.936	0.499	1.432	1.479	1.441	1.346	0.949	3.0	3.0
5_7_S335	0.873	1.206	0.796	0.616	0.683	1.263	0.851	0.788	0.407	3.0	3.5
5_8_S347	1.027	1.368	1.165	0.787	0.044	0.547	0.623	0.686	0.679	4.0	0.0
6_1_S358	1.319	1.556	1.250	0.578	0.662	0.542	0.489	0.475	0.744	3.0	3.5
6_2_S369	0.964	1.021	0.884	0.446	0.775	1.507	0.741	0.654	0.614	2.0	0.0
6_3_S288	1.076	1.397	1.006	0.416	0.590	0.655	0.494	0.662	0.322	3.0	0.0
6_4_S300	0.990	1.292	1.209	0.385	0.455	0.760	0.692	0.640	0.408	3.0	0.0
6_5_S312	1.015	1.498	1.150	0.715	0.781	0.926	1.167	0.798	0.677	3.0	3.5
6_6_S324	1.070	1.171	1.351	0.537	1.078	1.436	1.117	1.117	0.815	4.0	0.0
6_7_S336	1.000	1.399	1.108	0.687	0.471	0.271	0.422	0.500	0.501	4.0	0.0
6_8_S348	0.922	1.324	1.107	0.298	1.163	1.448	1.316	0.838	0.106	3.0	3.0
7_1_S359	2.480	3.102	2.832	1.327	2.534	3.031	2.778	2.905	1.172	2.0	3.0
7_2_S370	1.157	2.000	1.449	0.716	1.355	2.041	1.562	1.462	0.839	4.0	3.5
7_3_S289	1.356	2.150	1.338	1.033	0.960	1.260	1.349	1.344	0.815	4.0	4.0
7_4_S301	1.532	2.079	1.523	0.991	1.187	2.136	1.690	1.606	0.834	4.0	4.0
7_5_S313	0.925	1.456	0.908	0.471	0.806	1.253	0.908	1.110	0.591	4.0	4.0
7_6_S325	1.523	2.193	1.798	0.822	1.197	1.610	1.337	1.068	0.930	4.0	4.0
7_7_S337	1.579	2.324	1.571	0.781	1.531	1.705	1.694	1.676	0.971	4.0	0.0
7_8_S349	1.771	2.467	2.023	1.237	1.472	2.101	1.739	1.845	1.130	5.0	4.5
8_1_S360	0.904	1.204	1.062	0.440	1.372	1.785	1.357	1.346	1.060	5.0	5.0
8_2_S371	1.332	1.558	1.344	0.955	1.492	1.946	1.598	1.467	0.894	5.0	4.5
8_3_S290	0.552	0.989	0.783	0.412	0.918	1.240	0.801	0.877	0.715	4.0	4.0
8_5_S302	1.176	1.393	1.280	0.745	0.748	0.809	1.241	1.102	0.986	4.0	4.0
8_6_S314	0.982	1.290	1.000	0.549	0.925	1.246	0.508	0.868	0.683	4.0	4.0
8_7_S326	0.696	0.698	1.084	0.135	0.502	0.582	0.830	0.215	0.281	3.0	3.0
8_8_S338	0.832	0.960	0.689	0.454	1.071	1.753	1.269	0.610	0.302	4.0	4.5

9_1_S350	1.349	1.809	1.288	0.640	0.774	0.693	0.743	1.393	0.576	5.0	4.0
9_2_S361	1.109	1.251	1.059	0.582	0.990	1.794	1.364	1.186	0.719	4.0	4.5
9_3_S372	0.787	1.092	0.755	0.444	0.711	1.231	0.654	0.526	0.342	4.0	3.5
9_4_S291	1.621	2.367	1.555	0.629	1.407	2.141	1.618	1.509	0.570	3.0	3.5
9_5_S303	1.052	1.261	1.397	0.299	0.629	1.199	0.554	0.272	0.087	4.0	3.5
9_7_S315	1.556	2.258	1.651	0.821	2.026	2.876	1.942	1.820	1.052	4.0	3.5
9_8_S327	0.479	0.951	0.751	0.334	0.424	0.779	0.736	0.647	0.326	4.0	4.5
